# Activation of PAR4 Upregulates p16 through Inhibition of DNMT1 and HDAC2 Expression via MAPK Signals in Esophageal Squamous Cell Carcinoma Cells

**DOI:** 10.1155/2018/4735752

**Published:** 2018-09-30

**Authors:** Ming Wang, Shuhong An, Diyi Wang, Haizhen Ji, Xingjing Guo, Zhaojin Wang

**Affiliations:** ^1^Department of Human Anatomy, Taishan Medical University, 2 Ying Sheng Dong Lu, Taian 271000, China; ^2^Department of Pathology, Affiliated Hospital of Taishan Medical University, Taian 271000, China; ^3^Department of Physiology, Taishan Medical University, Taian 271000, China

## Abstract

A previous study showed that a downexpression of protease-activated receptor 4 (PAR4) is associated with the development of esophageal squamous cell carcinoma (ESCC). In this study, we explored the relationship between PAR4 activation and the expression of p16, and elucidated the underlying mechanisms in PAR4 inducing the tumor suppressor role in ESCC. ESCC cell lines (EC109 and TE-1) were treated with PAR4-activating peptide (PAR4-AP). Immunohistochemistry for DNA methyltransferase 1 (DNMT1) and histone deacetylase 2 (HDAC2) was performed in 26 cases of ESCC tissues. We found that DNMT1 and HDAC2 immunoreactivities in ESCC were significantly higher than those in adjacent noncancerous tissues. PAR4 activation could suppress DNMT1 and HDAC2, as well as increase p16 expressions, whereas silencing PAR4 dramatically increased HDAC2 and DNMT1, as well as reduced p16 expressions. Importantly, the chromatin immunoprecipitation-PCR (ChIP-PCR) data indicated that treatment of ESCC cells with PAR4-AP remarkably suppressed DNMT1 and HDAC2 enrichments on the p16 promoter. Furthermore, we demonstrated that activation of PAR4 resulted in an increase of p38/ERK phosphorylation and activators for p38/ERK enhanced the effect of PAR4 activation on HDAC2, DNMT1, and p16 expressions, whereas p38/ERK inhibitors reversed these effects. Moreover, we found that activation of PAR4 in ESCC cells significantly inhibited cell proliferation and induced apoptosis. These findings suggest that PAR4 plays a potential tumor suppressor role in ESCC cells and represents a potential therapeutic target of this disease.

## 1. Introduction

Protease-activated receptors (PARs), a superfamily of G-protein-coupled receptors that are activated by thrombin, have been perceived in multiple cells affiliated with inflammatory reactions, such as macrophages, neutrophils, and mast cells [1]. The recent detection of PARs on various cancer cells suggests that PARs might be involved not only in inflammation, but also in the development of cancers [2]. Several studies suggest that PARs play roles in cancer progression including tumor growth, invasion, migration, survival, and metastasis [3, 4]. Studies investigating the role of PAR4 in cancer have had conflicting results, as they were found to be overexpressed in several malignant tumors and implicated in tumor growth and cancer metastasis [4–6]. However, other studies showed a downexpression of PAR4 in esophageal, lung, and gastric cancers [7–9]. Recently, studies demonstrated that mice with knockdown PAR4 gene could accelerate tumor growth [10] and reduce cardiomyocyte apoptosis [11]. PAR4 is highly expressed in human esophageal squamous epithelial cells [9] and frequently downregulated in esophageal squamous cell carcinoma (ESCC) tissue, which is partly the result of the hypermethylation of the PAR4 promoter [8]. However, the role of PAR4 in the progress of ESCC has not been defined.

ESCC is one of the world's most aggressive types of malignancy with a poor prognosis [12]. Tobacco smoking is one of major risk factors for ESCC [13]. Exposure to carcinogens of tobacco smoke may result in the methylation of PAR4 gene, which is considered to be involved in carcinogenesis [14, 15]. p16, the tumor suppressor gene, is involved in the pathogenesis of esophageal cancer by influencing the cyclin kinase inhibitor cascade and DNA mismatch repair processes [16]. The promoter methylation inactivation of p16 gene can increase the risk of ESCC [17]. Previous studies have demonstrated that DNA methyltransferase 1 (DNMT1) is required for the maintenance of DNA methylation and the deactivation of p16 by DNMT1-mediated methylation that may lead to the development of ESCC [18]. At promoters, DNA methylation generally precludes transcription directly by blocking the binding of transcriptional activators or indirectly through the recruitment of methyl-binding proteins and corepressor complexes containing histone deacetylases (HDACs), which cooperatively facilitate the formation of heterochromatin [19].

In the present study, the association between the activation of PAR4 and expression of p16 protein and gene, as well as the enrichments of DNMT1 and HDAC2 on the p16 promoter, was examined by Western blotting, quantitative real-time PCR (qRT-PCR), and chromatin immunoprecipitation-PCR (ChIP-PCR) methods to identify the potentially diagnostic or therapeutic value of PAR4 in ESCC.

## 2. Materials and Methods

### 2.1. ESCC Cell Lines and Reagents

Human ESCC cell lines (EC109 and TE-1) were obtained from the Cell Bank of the Chinese Academy of Sciences (Shanghai) or National Infrastructure of Cell Line Resource (Beijing). The following reagents were used in this study: the selective PAR4-activating peptide (PAR4-AP) from Bachem; PAR4 control peptide from Tocric Bio-technology; PD98059 (an extracellular regulated protein kinase 1/2, ERK1/2, inhibitor), SB203580 (a p38 mitogen-activated protein kinase (MAPK), p38, inhibitor), and *t*-butylhydroquinone (tBHQ, an ERK1/2 activator) from Santa Cruz Biotechnology; and U-46619 (an ERK1/2 and p38 activator) from Millipore. This study protocol was approved by the Ethics Committee of the Taishan Medical University.

### 2.2. Drug Administration

Cultured ESCC cell lines were stimulated with PAR4-AP at a concentration of 100 *μ*M for 1, 2, 6, 12, and 24 h. Cells with PAR4 control peptide (100 *μ*M) treatment were used as control. To assess the possible effects of ERK1/2 and p38 on the regulation of p16, DNMT1, and HDAC2 expression following PAR4-AP stimulation, PD98059 (10 *μ*M), SB203580 (10 *μ*M), tBHQ (50 nM), or U-46619 (10 nM) was added to six-well plates 60 min prior to the addition of PAR4-AP. Cells stimulated with the PAR4 control peptide without ERK1/2 or p38 inhibitors or activators were used as controls.

### 2.3. Immunohistochemistry Analysis

Twenty-six ESCC tissues and their corresponding nearby nontumorous tissues were obtained from the Affiliated Hospital of Taishan Medical University, with the approval of the Local Research Ethics Committee. ESCC specimens were fixed in 10% buffered formalin. Paraffin sections were stained with anti-DNMT1 and HDAC2 (Abcam), then counterstained with hematoxylin. The immunoreaction score was then calculated by multiplying the percentage and intensity scores.

### 2.4. siRNA Transfection

siRNA targeting human PAR4 was synthesized by Sigma-Aldrich. A scrambled duplex siRNA was used as the negative control. ESCC cells were plated at 2 × 10^5^/well in 6-well plates and incubated until they reached 50% confluency. Cells were transfected with PAR4-siRNA or the negative control siRNA at a final concentration of 50 nM with Lipofectamine 2000 Transfection Reagent (Invitrogen) according to the manufacturer's recommendations. After 6 h of transfection, the medium was replaced with RPMI-1640 medium containing 10% fetal bovine serum. Cells were then incubated for 72 h for RNA isolation and protein extraction.

### 2.5. Western Blot Analysis

ESCC cells were continuously stimulated with PAR4-AP at a concentration of 100 *μ*M for 1, 2, 6, 12, and 24 h. Then, cells were lysed, and protein was extracted. Protein lysate from each sample was separated electrophoretically in sodium dodecyl sulfate polyacrylamide gel and then transferred to polyvinylidene fluoride (PVDF) membranes. Western blot analyses were performed with anti-DNMT1, HDAC2, p16 (Abcam), PAR4 (Alomone Labs), phosphorylated-ERK1/2 (p-ERK1/2), phosphorylated-p38 (p-p38), and GAPDH (Cell Signaling Technology).

### 2.6. qRT-PCR Assay

Total RNA was isolated from ESCC cell lines using TRIzol reagent (Invitrogen). qRT-PCR was performed to measure the expression of p16 on the 7300 Real-Time PCR System (Applied Biosystems, CA, USA). The synthetic oligonucleotide primer sequences were as follows: DNMT1, 5′-CCT AGC CCC AGG ATT ACA AGG-3′(sense) and 5′-ACT CAT CCG ATT TGG CTC TTT C-3′ (antisense); HDAC2, 5′-TCC GCA TGA CCC ATA ACT TGC-3′ (sense) and 5′-CCG CCA GTT GAG AGC TGA C-3′ (antisense); p16, 5′-GTG TAT AGG GTC GGC CAT CAA-3′ (sense) and 5′-AGC AAA ACC AAC CTA TAC CG-3′ (antisense); *β*-actin, 5′-GTG TAT AGG GTC GGC CAT CAA-3′ (sense) and 5′-TTT GTT TGT GGT CTT GTC CAGT-3′ (antisense). A comparative cycle threshold fluorescence (ΔCt) method was used, and the relative transcript amount of the target gene was normalized to that of *β*-actin using the 2^−ΔΔCT^ method. The final results of the real-time PCR are expressed as the ratio of the test mRNA to the control. All PCR product sizes were confirmed by electrophoresis on a 1.5% agarose gel and visualization using ethidium bromide.

### 2.7. ChIP-PCR

ChIPs were performed for human ESCC cell lines (EC109 and TE-1) and were analyzed essentially according to the instructions of One-Day Chromatin Immunoprecipitation Kit (Millipore). The DNA precipitated by the target antibodies (DNMT1, Abcam, ab13537; HDAC2, Abcam, ab12169) was detected with qRT-PCR. The primer sequences of the ChIP-qPCR reaction were as follows: p16-1, 5′-CTG CTC TTA TAC CAG GCA ATG TA-3′ (sense) and 5′-CCT GTA CGA CTA GAA AGT GTC CC-3′ (antisense); p16-2, 5′-TTT CCC TAT GAC ACC AAA CAC C-3′ (sense) and 5′-CCG CGA TAC AAC CTT CCT AAC-3′ (antisense); p16-3, 5′-CCT CCT TGC GCT TGT TAT ACT CT-3′ (sense) and 5′-CCC TCC ACC ACC CTC ACT TA-3′ (antisense). Control PCRs for each antibody immunoprecipitation were performed using primers for GAPDH and IGFBP3 (DNMT1) or CD4 and von Willebrand factor (HDAC2) as negative and positive controls dependent on the antibody used. All PCR product sizes were confirmed by electrophoresis. Each ChIP experiment was done in triplicate and repeated at least three times.

### 2.8. Cell Viability and Apoptosis Assay

Cell growth was detected by 3-(4,5-dimethylthiazol-2-yl)-2,5-diphenyltetra-zolium bromide (MTT) assay. MTT solution (0.5 mg/mL) was added to the cells and then the cells were cultured continuously for 4 h. Each sample was mixed and the optical density was measure at 570 nm. Apoptosis was assessed by an Andy Fluor 488 Annexin V and PI Apoptosis Kit (GeneCopoeia) and flow cytometry was performed using FACSCalibur (BD Biosciences) according to the manufacturers' instructions. All experiments were performed in triplicate.

### 2.9. Real-Time Cell Analysis (RTCA)

Cells were seeded in separate electronic 16-well plates with an integrated microelectronic sensor array in 100 *μ*l of suitable culture medium (RTCA DP, ACEA Biosciences). After 24 h, PAR4-AP was added to a total volume of 100 *μ*l at a concentration of 100 *μ*M. Cell proliferation and survival were monitored in real-time by measuring the cell-to-electrode responses of the seeded cells. The cell index (CI) was calculated for each E-plate well by RTCA Software. The graphs are generated in real time by the xCELLigence system.

### 2.10. Statistical Analysis

All experiments were repeated independently, at least three times. Values are expressed as mean ± SEM, and results were analyzed using an ANOVA followed by a Bonferroni test for comparison among groups. Significance was defined as *P* values < 0.05.

## 3. Results

### 3.1. Expression of DNMT1 and HADC2 in Human ESCC Tissues

**Figure 1:**
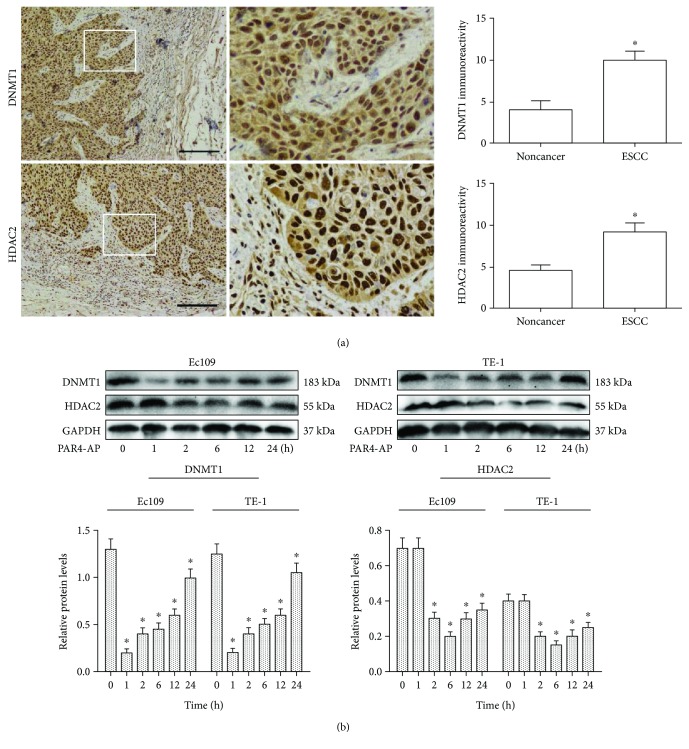
Immunohistochemical staining for DNMT1 and HDAC2 in ESCC tissues and effects of PAR4-AP on their expressions in ESCC cell lines after PAR4-AP treatment. (a) Representative immunostaining for DNMT1 and HDAC2 in ESCC tissues and adjacent noncancerous tissues. Sections were counterstained with hematoxylin. Scale bar: 100 *μ*m. Graphs showing DNMT1 and HDAC2 immunostaining in ESCC tissues and adjacent noncancerous tissues. The data are expressed as the mean ± SEM. ^∗^*P* < 0.01 (Student's *t*-test). Immunoreactivity was calculated by multiplying the percentage and intensity scores. (b) Examples of Western blotting showing DNMT1 and HDAC2 expressions in ESCC cell lines (EC109 and TE-1) with PAR4-AP treatment at 0, 1, 2, 6, 12, and 24 h, respectively. The mean optic densities of the proteins were calculated by normalizing to GAPDH. The data are presented as the mean ± SEM (*n* = 3), ^∗^*P* < 0.05 versus controls.

Immunoreactivity for DNMT1 and HDAC2 was analyzed in 26 paired human ESCC and adjacent nontumorous tissues. DNMT1 and HDAC2 were mainly in nuclear staining in ESCC tissues and adjacent nontumorous tissues. Levels of both protein immunoreactivities in ESCC tissues were significantly higher than those in adjacent nontumorous tissues (Figure 1(a)). These suggest that interaction between DNMT1 and HDAC2 might be involved in ESCC carcinogenesis [20].

### 3.2. The Effect of PAR4 Activation on DNMT1, HDAC2, and p16 in ESCC Cells

**Figure 2:**
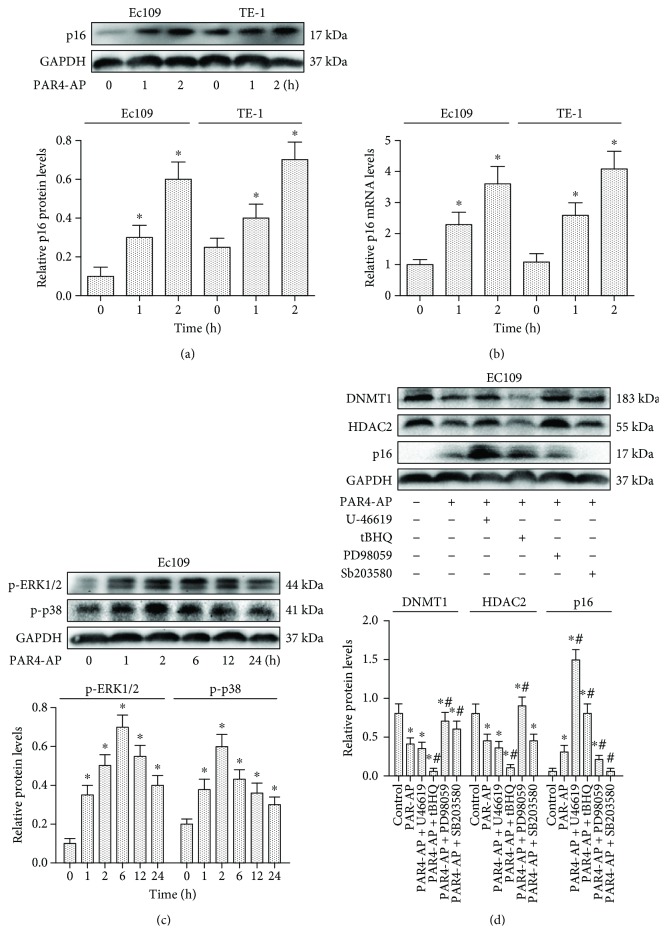
Effect of MAPK on DNMT1, HDAC2, and p16 expression by PAR4-AP in human ESCC cells. (a) Western blotting showing p16 protein levels in ESCC cell lines (EC109 and TE-1) with PAR4-AP treatment at 0, 1, and 2 h, respectively. (b) RT-PCR analysis showing p16 gene levels in ESCC cell lines (EC109 and TE-1) with PAR4-AP treatment at 0, 1, and 2 h, respectively. The results were calculated by normalizing to *β*-actin in the same sample with the ΔCt method. The changes in the relative mRNA levels are expressed as fold changes compared with the controls. (c) Western blotting showing p-ERK1/2 and p-p38 expression in ESCC cells with PAR4-AP treatment at 0, 1, 2, 6, 12, and 24 h, respectively. (d) Western blotting analyses for DNMT1, HDAC2, and p16 protein expressions in ESCC cells following treatment with PAR4-AP for 2 h and pretreatment with U-46619 (an activator of ERK1/2 and p38), tBHQ (an activator of ERK1/2), PD98059 (an inhibitor of ERK1/2), or SB203580 (an inhibitor of p38) for 60 min. The mean optic densities of the proteins were calculated by normalizing to GAPDH. All values are expressed as the means ± SEMs (*n* = 3). ^∗^*P* < 0.05 versus controls; ^#^*P* < 0.05 versus PAR4-AP-only groups.

The expression of DNMT1 and HDAC2 in ESCC cell lines (EC109 and TE-1) was assessed by Western blot after treatment with PAR4-AP for 1, 2, 6, 12, and 24 h, respectively. As shown in Figure 1(b), DNMT1 and HDAC2 levels were downregulated by PAR4-AP treatment. Meanwhile, PAR4-AP treatment of ESCC cells significantly increased p16 protein and mRNA levels compared with the control groups (Figures 2(a) and 2(b)). These results suggested that the upregulation of p16 protein and gene expression by PAR4 might be associated with suppression of DNMT1 and HDAC2 [21].

### 3.3. Effect of MAPK on DNMT1, HDAC2, and p16 Expression by PAR4-AP in ESCC Cells

To assess the possible effects of MAPK on the regulation of DNMT1, HDAC2, and p16 expression by PAR4, ESCC cells were treated with PAR4-AP for 2 h and pretreated with U-46619 (ERK1/2 and p38 activator), tBHQ (ERK1/2 activator), PD98059 (ERK1/2 inhibitor), or SB203580 (p38 inhibitor) for 60 min. Activation of PAR4 increased p-ERK1/2 and p-p38 expression in ESCC cells after PAR4-AP treatment (Figure 2(c)). Compared with PAR4-AP alone, PAR4-AP with U-46619 or tBHQ, activators for ERK1/2 and p38, could induce a decrease of DNMT1 and HDAC2 protein levels to much lower levels, which in turn markedly increased the expression of p16 protein (Figure 2(d)). Meanwhile, PD98059 or SB203580, inhibitors for ERK1/2 and p38, partially or completely blocked the increase of p16 protein expression, which in turn markedly reversed the downexpression of DNMT1 and HDAC2 proteins, compared with PAR4-AP-only groups (Figure 2(d)). These results indicated that the effect of PAR4-AP on DNMT1, HDAC2, and p16 expression is associated with MAPK signal pathways [22].

### 3.4. PAR4 Increased p16 Gene Expression by Attenuating DNMT1 and HDAC2 Enrichments on the p16 Promoter

**Figure 3:**
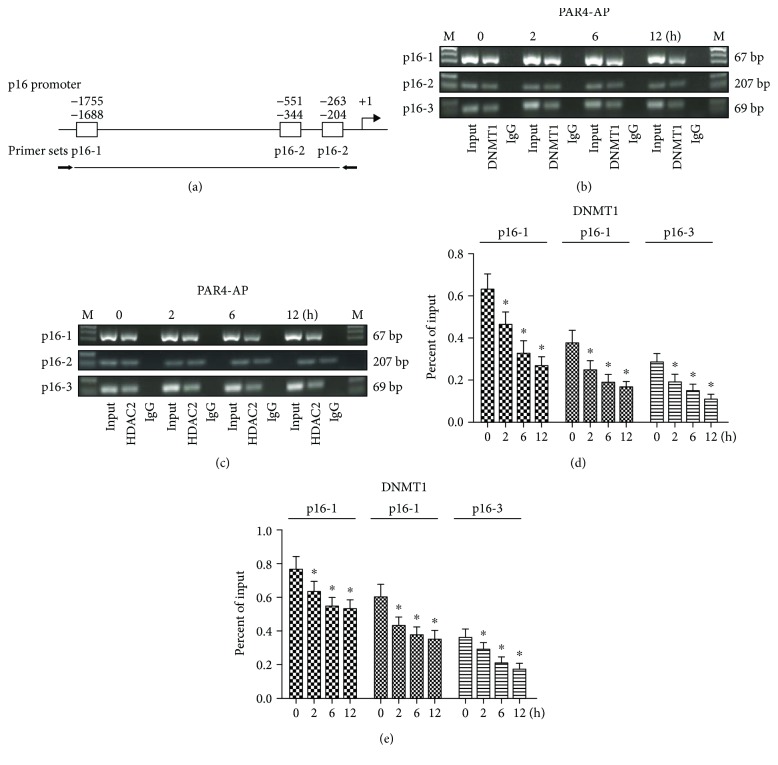
PAR4-AP suppressed DNMT1 and HDAC2 enrichments on the p16 promoter in ESCC cells. (a) Schematic representation of the p16 promoter region. P16-1, p16-2, and p16-3 indicate the locations of primers on the p16 promoter. (b, c) ChIP assays were performed for the p16 promoter in ESCC cells using antibodies to DNMT1 and HDAC2 after treatment of PAR4-AP at 2, 6, and 12 h. Normal rabbit IgG served as a negative control, and input chromatin (samples without immunoprecipitation (IP)) served as a positive control. (d, e) ChIP-qPCR assays to evaluate DNMT1 and HDAC2 enrichments in the p16 promoter region of ESCC cells with treatment of PAR4-AP at 2, 6, and 12 h. The data are expressed as the mean ± SEM (*n* = 3). ^∗^*P* < 0.05 versus controls.

To evaluate the effect of PAR4 on the physical interaction between DNMT1 and HDAC2 on promoter regions of p16, ChIP-PCR experiments for human ESCC cells were performed after treatment with PAR4-AP for 2, 6, and 12 h. We designed a series of primer coordinates to the three regions in the p16 promoter for ChIP-PCR assays (Figure 3(a)). p16-1, p16-2, and p16-3 are located upstream of the p16 promoter (−1755 bp, −551 bp and− 263 bp), representing the important regulatory regions of p16 gene. ChIP-PCRs in ESCC cell lines demonstrated that the p16 promoter regions (p16-1, p16-2, and p16-3) have less enrichments of DNMT1 and HDAC2 in ESCC cells after treatment with PAR4-AP for 2, 6, and 12 h, compared with controls (*P* < 0.05) (Figures 3(b)–3(e)). This result implicated that the promoting p16 transcription by activation of PAR4 might be associated with attenuating DNMT1 and HDAC2 enrichments on the p16 promoter.

### 3.5. DNMT1, HDAC2, and p16 Expression of ESCC Cell Lines following PAR4 Gene Knockdown

**Figure 4:**
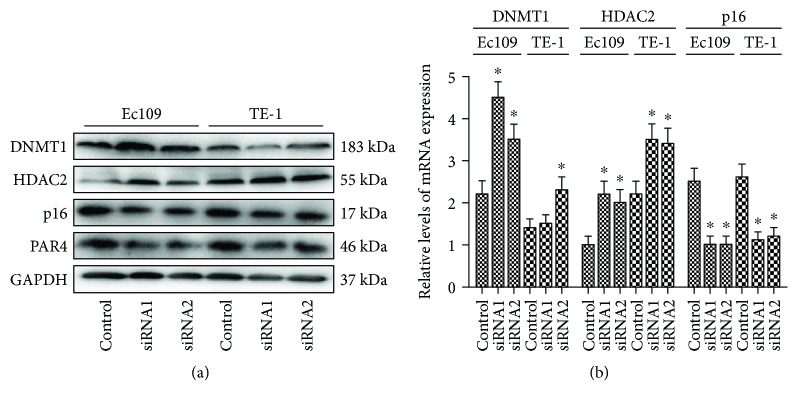
Silencing PAR4 induces DNMT1, HDAC2, and p16 expression in ESCC cells. (a) ESCC cells were transiently transfected with PAR4-siRNA1 or PAR4-siRNA2. Western blotting was performed to examine the expression of DNMT1, HDAC2, p16, and PAR4 at protein levels after transfection with PAR4-siRNA for 72 h. (b) qRT-PCR was performed to examine the expression of DNMT1, HDAC2, and p16 expression at mRNA levels after transfection with PAR4-siRNA for 72 h. The results were calculated by normalizing to *β*-actin in the same sample with the ΔCt method. The data are expressed as the mean ± SEM (*n* = 3). ^∗^*P* < 0.05 versus controls.

Western blotting and qRT-PCR assay were used to determine the effects of PAR4-siRNA-mediated PAR4 silencing on DNMT1, HDAC2, and p16 expression of ESCC cell lines. The cells were cultured for 72 h subsequent to transfection of PAR4-siRNA. The results confirmed that the expression of PAR4 protein was inhibited by transfection of PAR4-siRNA (Figure 4(a)). The PAR4 gene knockdown was able to upregulate the levels of DNMT1 and HDAC2 proteins and genes and suppress p16 protein and gene expression following transfection of PAR4-siRNA with ESCC cells (*P* > 0.05) (Figures 4(a) and 4(b)).

### 3.6. Effects of PAR4 Activation on Cell Proliferation and Apoptosis in ESCC Cells

**Figure 5:**
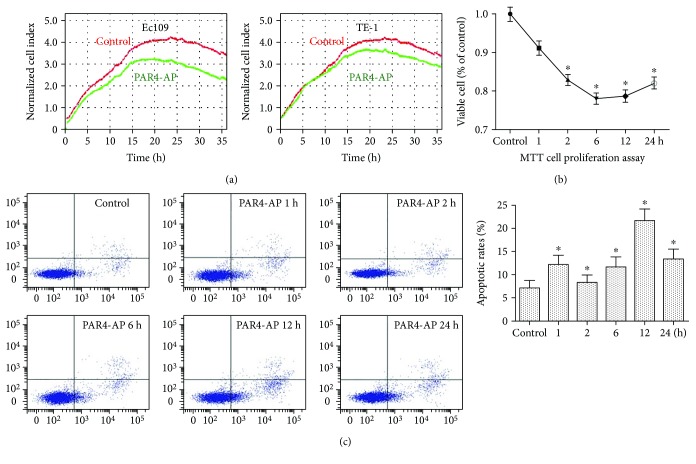
Effects of PAR4-AP on cell proliferation and apoptosis in ESCC cells. (a) RTCA was performed to evaluate the proliferation and viability of ESCC cells following continuous treatment with PAR4-AP. Green lines represent cell index (CI) treated with PAR4-AP, and red lines are CI treated with the PAR4 control peptide. (b) The cell viability of ESCC cells was measured by MTT assay following 1, 2, 6, 12, and 24 h of treatment with PAR4-AP, respectively. (c) Cells were stained with Andy Fluor 488 Annexin V and PI and analyzed by flow cytometry following treatment with PAR4-AP. The ratio of apoptosis in ESCC cells was detected by flow cytometry following 1, 2, 6, 12, and 24 h of treatment with PAR4-AP, respectively. The data are expressed as the mean ± SEM (*n* = 3). ^∗^*P* < 0.05 versus controls.

In order to determine the effect of PAR4-AP on the growth of ESCC cell lines, ESCC cells were treated with PAR4-AP and cell proliferation was assessed using RTCA and MTT assay. RTCA proliferation assay demonstrated that the cell index decreased in a time-dependent manner following PAR4-AP treatment and was significantly lower in PAR4-AP groups when compared with the control group following treatment for 24 h (Figure 5(a)). Similarly, MTT assay analysis demonstrated that the viability of ESCC cells decreased in a time-dependent manner following PAR4-AP treatment (Figure 5(b)). The effect of PAR4 activation on the apoptosis of ESCC cells treated with PAR4-AP was detected by flow cytometry. Graphical representation of the apoptosis assay showed that treatment with PAR4-AP led to an increase of apoptosis (Figure 5(c)). The results indicated that the apoptosis rate of ESCC cells was significantly increased in a time-dependent manner following PAR4-AP treatment.

## 4. Discussion

Our study demonstrated that the treatment with PAR4-AP inhibited the proliferation of ESCC cells, upregulated p16, and reduced DNMT1 and HDAC2 expression in ESCC cells. The ChIP study revealed that activation of PAR4 suppressed the enrichments of DNMT1 and HDAC2 on the p16 promoter region. These effects were associated with MAPK signals that were induced by PAR4 activation. These findings provide evidence that the inhibited proliferation of ESCC cells associated with PAR4-AP may be involved in the promotion of p16 transcription through suppressing DNMT1 and HDAC2 expression via MAPK signals in ESCC cells.

p16 is an important tumor suppressor protein that plays essential roles during cell proliferation through regulating the expression of several genes [23]. The p16 gene encodes a p16 protein that binds competitively to CDK4 and, during G1 phase, inhibits the interaction of CDK4 and cyclin D1 to stimulate passage through the cell cycle [24–26]. The p16 protein is often highly expressed in senescent cells in culture and is inactivated in a variety of human cancers, and the p16 could enhance the apoptotic functions of p53 through DNA-dependent interaction [27]. Our results showed that activation of PAR4 could inhibit proliferation and induce apoptosis, as well as upregulate p16 expression following PAR4-AP treatment of ESCC cells. Therefore, it is possible that the inhibition of proliferation and increase of apoptosis evoked by PAR4 activation is closely related to p16 gene transcription in ESCC cells.

It is believed that DNMT1 is required for the deactivation of p16 by DNMT1-mediated methylation that may lead to the development of ESCC [18]. Growing evidence shows that overexpressed HDACs are associated with tumorigenesis in ESCC [28]. Previous studies showed that HDACs form a complex with DNMT1 and that the protein stability of DNMT1 is regulated by posttranslational modifications of acetylation and ubiquitination [29]. The inhibition of DNMT1 might reduce DNA methylation in the p16 promoter and increase p16 expression [30]. HDAC2 inactivation significantly reduced G1-S cell cycle arrest, restored activity of p16, and promoted apoptosis [29]. In the present study, the activation of PAR4 was able to increase p16 expression and decrease DNMT1 and HDAC2 expression in ESCC cells. Therefore, it seems more likely that the effect of PAR4 on DNMT1 and HDAC2 expression results in promoting p16 gene transcription [18, 31]. These results suggested that PAR4 was able to upregulate p16 levels through inhibition of DNMT1 and HDAC2 expression in ESCC cells.

To understand the relationship between the activation of PAR4 and the physical interplay of DNMT1 with HDAC2 on the p16 promoter, we performed ChIP-PCR to investigate the enrichments of DNMT1 and HDAC2 on the p16 promoter in ESCC cells. The results demonstrated that activation of PAR4 could decrease the enrichments of DNMT1 and HDAC2 on the p16 promoter in ESCC cells. Suppressing DNMT1 could lead to the activation of the esophageal suppressor gene p16 [18]. DNMT1 usually forms a corepressor complex with HDACs and represses transcription [19, 31]. Therefore, it is likely that the PAR4 that led to decreased DNMT1 and HDAC2 binding to the p16 promoter of ESCC cells was likely involved in the activating transcription of this gene. The decreased DNMT1 and HDAC2 enrichments in ESCC cells after PAR4-AP treatment provided a novel molecular mechanism for promoting p16 gene transcription in ESCC cells.

In the present study, PAR4 activation increased p-ERK1/2 and p-p38 expression in ESCC cells after PAR4-AP treatment. ERK1/2 and p38 activators enhanced the effects of PAR4 activation on DNMT1, HDAC2, and p16 expression, whereas ERK1/2 and p38 inhibitors reversed these effects. Several studies have supported the idea that PAR4 activation is involved in the initiation of the MAPK signal pathways [32–34]. Previous reports implicated the involvement of the MEK/ERK pathway in the reduction of DNMT1 expression and function [35, 36]. Activation of the ERK1/2 signaling pathway evoked by PARs induces the activation of the transcriptional factor CREB, which in turn leads to gene expression [37]. CREB-binding protein (CBP), a histone acetyltransferase, could bind to CREB and enhance p16 expression [38]. Activation of the p38 pathway induces hepatocellular carcinoma cell apoptosis through suppression of DNMT1 expression [39]. It also suppresses the growth of human leukemia cells by downregulating HDAC2 expression and activity [40]. Because of the effect of MAPK on the expressions of DNMT1, HDAC2, and p16 induced by PAR4-AP, it is likely that the inhibitions of DNMT1 and HDAC2 induced by PAR4 activation are involved in the expression of p16 via MAPK signals in ESCC cells. The increase in p16 mRNA and protein levels in ESCC cells after PAR4-AP treatment provides a molecular mechanism for PAR4 in the regulation of ESCC carcinogenesis.

## 5. Conclusion

To our knowledge, we are the first to report that PAR4 activation could inhibit the viability and induce the apoptosis of ESCC cells through suppressing enrichments of DNMT1 and HDAC2 at the p16 promoter via MAPK signals. The results of this study provide new insight into the mechanisms of the promoting p16 gene transcription by PAR4 activation in ESCC cells. Therapeutically, PAR4 may be a potent target for the treatment of ESCC.

## Data Availability

The data used to support the findings of this study are available from the corresponding author upon request.
